# Measurement of Serum Zinc Levels Following the Use of Oral Medications and Supplements and Their Effects on Semen Characteristics in Male Infertility Patients

**DOI:** 10.7759/cureus.105863

**Published:** 2026-03-25

**Authors:** Hatsuki Hibi, Mikiko Tokoro, Shota Takizawa, Yoshimasa Asada

**Affiliations:** 1 Urology Department, Kyoritsu General Hospital, Nagoya, JPN; 2 Research Department, Asada Institute for Reproductive Medicine, Nagoya, JPN; 3 Obstetrics and Gynecology Department, Asada Ladies Clinic, Nagoya, JPN

**Keywords:** assisted reproductive technology (art), male infertility, semen quality, serum zinc concentration, testosterone

## Abstract

Background and aim

Many male infertility patients take empiric oral medications and/or zinc-containing supplements. Zinc is an important trace element, and its importance for male infertility has been reported. To evaluate their impact, zinc concentrations (Zc) were assessed, and the relationship between semen findings, infertility treatment, and outcome with assisted reproductive technology (ART) was examined.

Methods

Of the 4601 subjects who visited our male infertility unit between August 2020 and December 2024, 4389 were eligible, excluding those who were single, had not undergone semen analysis, and those taking both medication and supplements.

Results

Median serum Zc was 84.0 (75.0, 94.0) μg/dL. A total of 1209 participants (27.5%) were taking medications or supplements. Although differences in Zc were observed depending on the time of blood retrieval, no correlation was found between Zc and sperm count or motility. There was also no significant difference in Zc between different infertility diagnoses or between groups not taking oral medication and those taking other prescribed medications, herbal medicines, or zinc-containing supplements. As expected, the pregnancy rate was higher in those with normal semen findings, and no correlation was found between pregnancy and serum Zc.

Conclusions

This large cross-sectional study did not find any correlation between serum Zc and sperm parameters. Furthermore, no significant differences in Zc or pregnancy rates were observed with the intake of various oral medications or supplements.

## Introduction

Zinc is the second most abundant trace element in humans and is a cofactor for over 300 enzymes. Since zinc cannot be stored in the human body, regular dietary intake is required. Protein and nucleic acid synthesis enzymes require zinc for catalytic activity. Therefore, zinc plays an important role in growth and tissue repair. Zinc is also important for hormone production, which may be related to semen parameters of male infertility [1,2]. Zinc plays an important role in membrane stabilization and antioxidant activity, and it maintains sperm viability by inhibiting DNases [3]. Many male infertility patients take empiric oral medications and/or zinc-containing supplements. In this study, we retrospectively assessed the relationship among zinc concentrations (Zc), semen findings, and infertility treatment outcomes in a larger number of male infertility patients.

This study was previously presented at the 47th Annual Meeting of the European Society for Clinical Nutrition and Metabolism (ESPEN), Prague, Czech Republic, September 13-16, 2025.

## Materials and methods

Study design and participants

Of the 4601 subjects who visited our male infertility unit between August 2020 and December 2024, 4389 were eligible. Semen analysis was performed at the first visit. A blood sample was taken for Zc at the time of the patient examination, along with routine hormone and chromosome tests. Pregnancy was attempted following assisted reproductive technology (ART).

Inclusion and exclusion criteria

All patients who visited our male infertility clinic and agreed to a blood test were asked to participate in this study. A total of 212 subjects were single, had not undergone semen analysis, or were taking both oral medication and supplements, and were excluded from this study.

Statistical analysis

Continuous variables were expressed as medians with interquartile ranges. Comparisons between two groups were performed using the Mann-Whitney U test. Comparisons of serum Zc and age among three or more groups were conducted using Steel’s multiple comparison test. Correlations between continuous variables were assessed using Spearman’s rank correlation. Trends were evaluated using the Cochran-Armitage trend test. Confidence intervals (CI) for proportions were calculated using the exact binomial (Clopper-Pearson) method. For continuous variables, 95% CI was estimated using a bootstrap approach. All statistical tests were two-sided, and p<0.05 was considered statistically significant. Statistical analyses were performed using R software (version 4.4.2; Vienna, Austria: R Foundation).

Statement of ethics

This was a retrospective observational study; the protocol for this research project, including its use of human subjects, was approved by a suitably constituted Ethical Committee of Kyoritsu General Hospital (approval number: 2025-01, approval date: April 02, 2025).

## Results

Patient characteristics were judged according to semen analyses. The number of subjects who showed normozoospermia was 2819, oligoasthenozoospermia 1411, cryptozoospermia 75, non-obstructive azoospermia 60, obstructive azoospermia 17, and ejaculatory dysfunction 7, respectively. Patient characteristics are shown in Table 1.

**Table 1:** Patient characteristics by diagnosis. The numbers in parentheses are shown as median (Q1, Q3). NOA: non-obstructive azoospermia; OA: obstructive azoospermia; OAT: oligoasthenozoospermia; EjD: ejaculatory dysfunction

Variables	Normozoospermia (n=2819)	Cryptozoospermia (n=75)	NOA (n=60)	OA (n=17)	OAT (n=1411)	EjD (n=7)
Age (years)	37 (33, 41)	36 (33, 42)	31 (29, 39)	38 (33, 44)	38 (34, 43)	36 (33, 44)
Spouse age (years)	36 (32, 40)	34 (31, 39)	30 (28, 36)	33 (29, 41)	37 (33, 41)	31 (28, 37)
Duration of infertility (years)	2.0 (1.0, 3.0)	2.0 (1.0, 3.0)	1.2 (1.0, 3.0)	2.5 (1.0, 5.0)	2.0 (1.0, 4.0)	2.0 (1.0, 3.0)
Testicular size; right (mL)	18.0 (16.0, 18.0)	11.0 (8.0, 14.0)	7.0 (4.0, 11.0)	18.0 (16.0, 20.0)	18.0 (16.0, 20.0)	20.0 (20.0, 20.0)
Testicular size; left (mL)	14.0 (14.0, 16.0)	11.0 (9.0, 14.0)	7.0 (4.0, 10.0)	16.0 (14.0, 18.0)	16.0 (14.0, 18.0)	20.0 (20.0, 20.0)
LH (mIU/mL)	4.60 (3.50, 5.90)	7.85 (4.90, 12.00)	10.15 (7.50, 14.80)	3.90 (2.20, 5.70)	4.90 (3.60, 6.70)	6.00 (4.20, 7.10)
FSH (mIU/mL)	4.5 (3.2, 5.9)	12.2 (6.3, 21.5)	25.3 (15.9, 39.3)	3.7 (3.0, 5.9)	5.0 (3.6, 7.0)	4.4 (3.6, 8.2)
Testosterone (ng/mL)	4.5 (3.5, 5.8)	4.5 (3.5, 6.0)	3.8 (2.9, 4.8)	4.3 (3.0, 5.1)	4.6 (3.5, 5.7)	5.6 (3.9, 6.8)
BMI (kg/m^2^)	23.1 (21.3, 25.1)	23.2 (21.2, 24.8)	23.4 (20.9, 25.5)	22.2 (21.5, 24.3)	23.1 (21.3, 25.3)	22.9 (21.0, 25.5)

Smoking, medication, and supplementation

A total of 2732 (62.2%) patients took no medication, whereas 1209 participants (27.5%) were taking medications or supplements. Smoking was observed in 448 participants (10.2%), and 647 (14.7%) participants took zinc-containing supplementation. Details of smoking, medications, and supplementation with each Zc value are shown in Table 2. The type of medications widely varied; the most used medications were anti-allergy medications, used by 135 (3.1%) participants. There were 85 (1.9%) participants receiving empiric medication such as herbal medicine, 73 (1.7%) for androgenic alopecia (AGA), 69 (1.6%) with an anti-dyslipidemia medication, 64 (1.5%) psychotropic medication, 57 (1.3%) anti-hypertensive medication, 37 (0.8%) anti-hyperuricemia medication, 27 (0.6%) diabetic, and 15 (0.3%) ulcerative colitis medication, respectively. Participants taking empiric medication had significantly lower Zc compared with those taking no medication (p<0.001).

**Table 2:** Subjects smoking, taking medication, or supplements. Zinc concentrations (Zc) are shown as median (Q1, Q3). Steel's test was used. Participants taking empiric medication had significantly lower Zc compared with those taking no medication (p<0.001).

Variables	Total=4389, n (%)	Zc (μg/dL)	p-Value
No medication/no smoking	2732 (62.2)	84.0 (76.0, 93.0)	-
No medication/smoking	448 (10.2)	84.0 (75.0, 95.0)	1
Supplementation	647 (14.7)	85.0 (76.0, 95.0)	0.645
Anti-allergic medication	135 (3.1)	81.0 (71.5, 91.0)	0.409
Empiric (ex. herbal medicine) medication	85 (1.9)	76.0 (68.0, 85.0)	<0.001
Androgenic alopecia (AGA) medication	73 (1.7)	84.0 (75.0, 98.0)	1
Anti-dyslipidemic medication	69 (1.6)	84.0 (74.0, 96.0)	1
Psychotropic medication	64 (1.5)	84.0 (75.0, 91.0)	1
Anti-hypertensive medication	57 (1.3)	82.0 (68.0, 89.0)	0.282
Anti-hyperuricemia medication	37 (0.8)	83.0 (73.0, 91.0)	1
Diabetic	27 (0.6)	83.0 (78.0, 93.5)	1
Ulcerative colitis medication	15 (0.3)	74.0 (71.5, 79.5)	0.086

Zinc concentration

**Figure 1:**
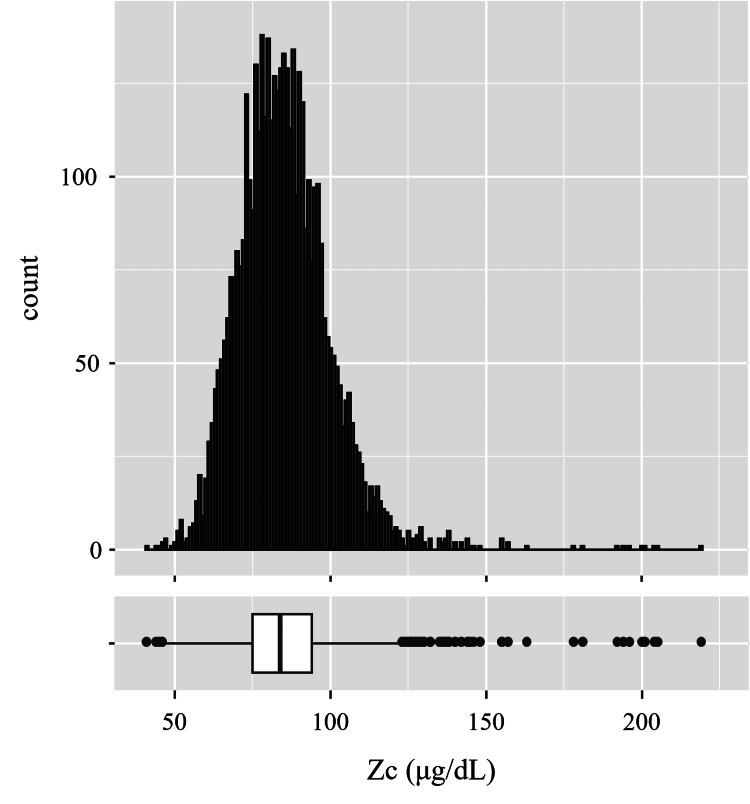
Histogram and box plot of zinc concentrations (Zc). Median serum zinc concentration was 84.0 (75.0, 94.0) μg/dL.

**Figure 2:**
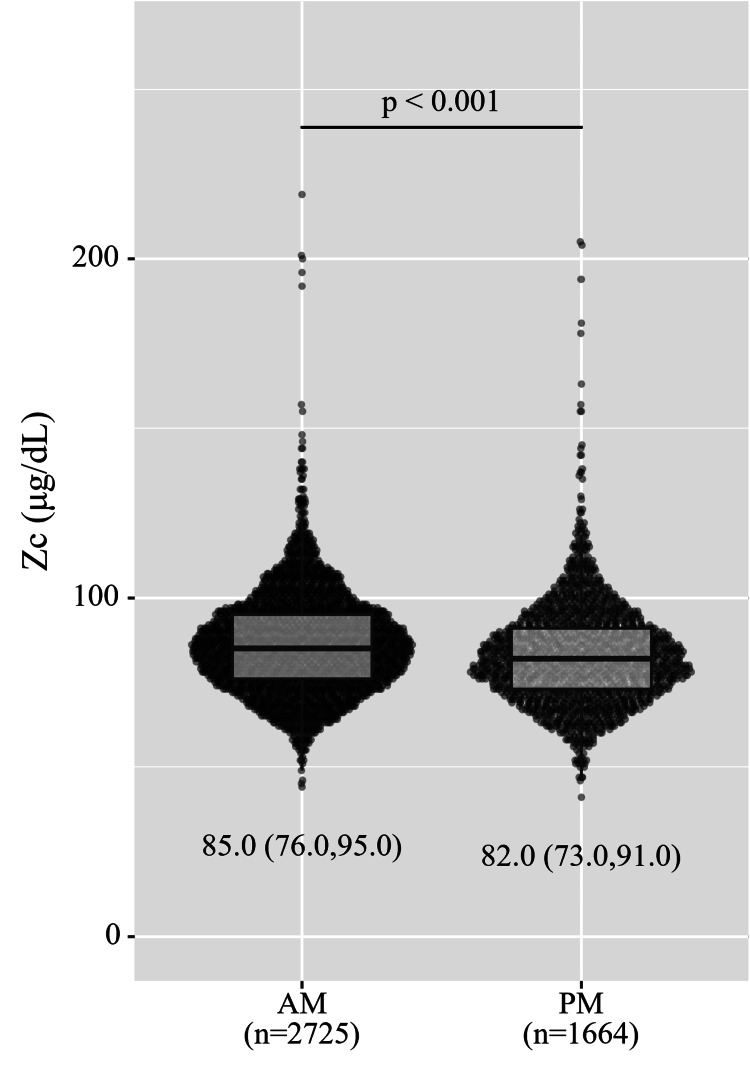
Time of day when blood was drawn. Zinc concentrations (Zc) are shown as median (Q1, Q3). Serum Zc was significantly lower in the afternoon than in the morning (p<0.001; Mann-Whitney U test was used, 95% CI was 85.0-86.0 μg/dL at AM, and 81.0-83.0 μg/dL at PM).

Overall median serum Zc was 84.0 (75.0, 94.0) μg/dL. The histogram and box plot of Zc are shown in Figure 1. According to the time of blood drawing, Zc was obviously lower in the afternoon (PM) than in the morning (AM). Zc are shown as median (Q1, Q3) (p<0.001, Mann-Whitney U test was used, 95% confidence interval was 85.0-86.0 μg/dL at AM, and 81.0-83.0 μg/dL at PM, using the Wilson score method) (Figure 2).

Zinc concentration and various parameters

**Figure 3:**
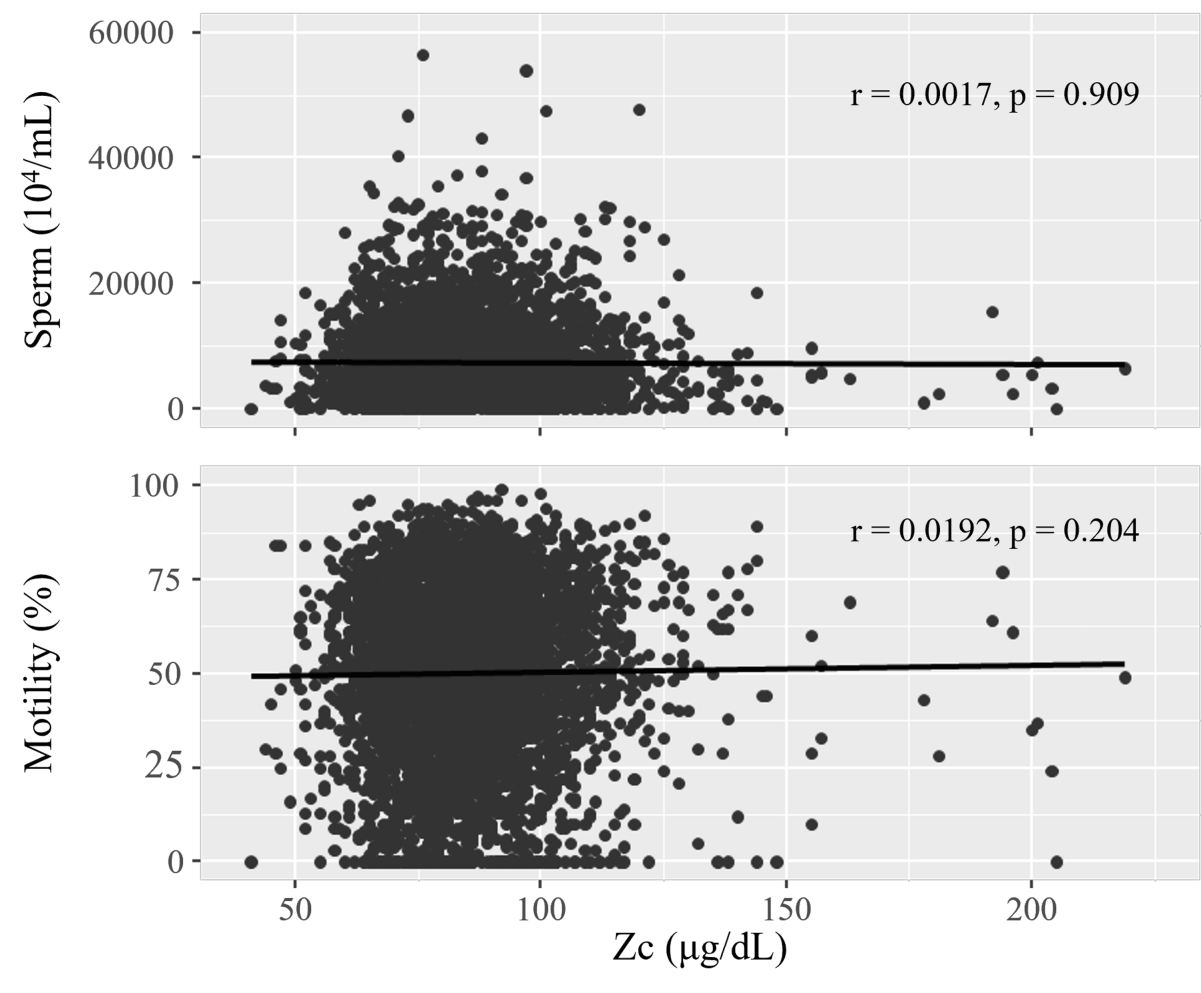
Zinc concentration and semen quality. Using Spearman’s rank correlation, no correlation was found between zinc concentrations (Zc) and sperm count (r=0.0017, 95% CI: -0.0293-0.0311) or motility (r=0.0192, 95% CI: -0.0111-0.0487).

Figure 3 shows the correlation between Zc and semen quality. Using Spearman’s rank correlation, no significant difference was found between Zc and sperm count or motility (95% confidence interval: -0.0293-0.0311 about sperm count and -0.0111-0.0487 about motility).

**Figure 4:**
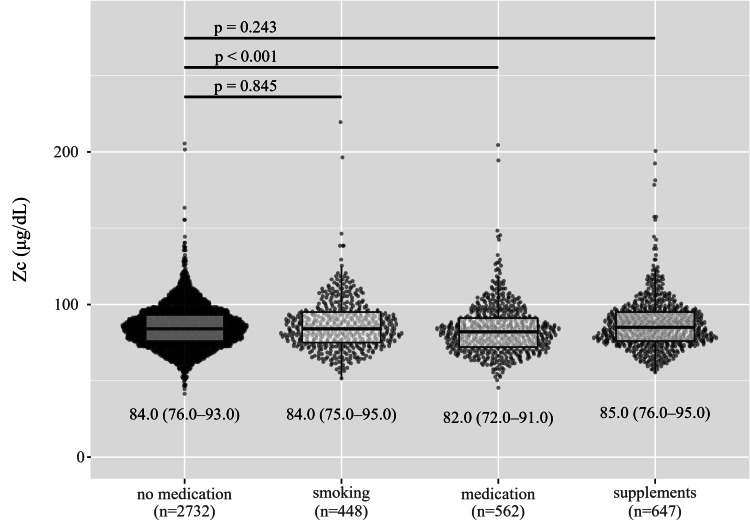
Zinc concentration and smoking, taking medication, or supplements. Zinc concentrations (Zc) are shown as median (Q1, Q3). Patients taking any medications had a lower Zc than those not taking any (p<0.001, using Steel’s multiple comparison test; 95% CI: 84.0-85.0). Zc showed no differences among no medication, smoking (95% CI: 83.0-86.0), and taking supplementation (95% CI: 83.0-87.0).

**Figure 5:**
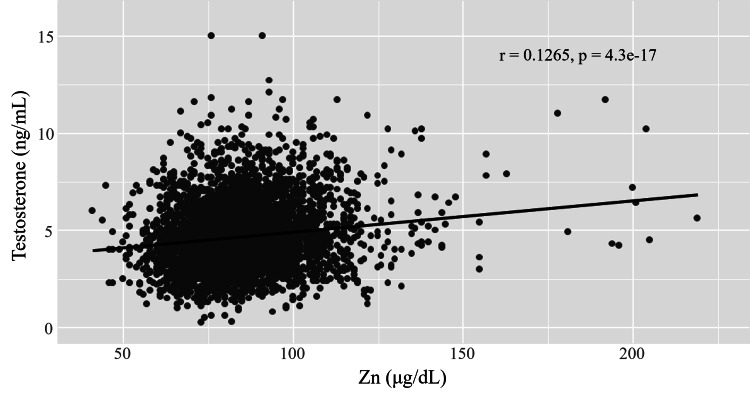
Zinc and testosterone. Zinc concentrations (Zc) and testosterone were positively correlated by using Spearman’s rank correlation (r=0.1265, 95% CI: 0.0973-0.1568).

Patients taking any medications (95% confidence interval: 80.0-83.0) had lower Zc than those not taking any medications (p<0.001, using Steel’s multiple comparison test, 95% confidence interval: 84.0-85.0) (Figure 4). Zc are shown as median (Q1, Q3). Medications included anti-allergic, empiric AGA, dyslipidemia, psychotropic, anti-hypertensive, anti-hyperuricemia, diabetic, and ulcerative colitis medication. However, Zc showed no differences among no medication, smoking (95% confidence interval: 82.0-87.0), and supplementation (95% confidence interval: 83.0-87.0). Figure 5 presents the relationship between Zc and testosterone. There was a positive correlation between Zc and testosterone using Spearman’s rank correlation (95% confidence interval: 0.116-0.145).

ART results

**Figure 6:**
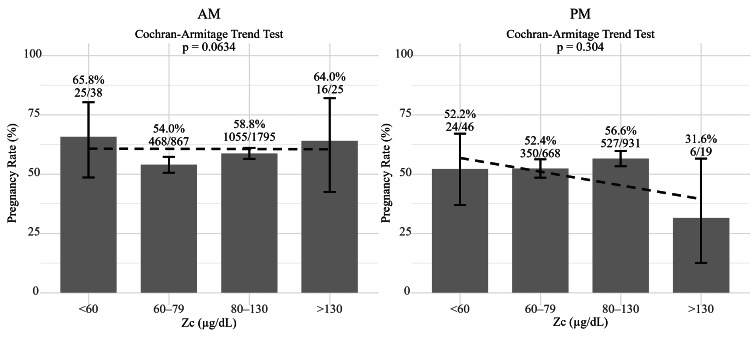
Zinc concentrations (Zc) and pregnancy rate; comparison based on time of day when blood was drawn. By using the Cochran-Armitage trend test, zinc concentrations did not affect pregnancy rates when blood drawing was performed in the morning or afternoon.

Overall, a clinical pregnancy was obtained in 2471/4389 (56.3%) following ART. Spouse age was not related to differences in patient Zc (Table 3). Spouse ages are shown as median (Q1, Q3). Although patients with high Zc showed significantly higher testosterone, Zc, based on the time of day when blood was drawn, was not associated with pregnancy following ART (using the Cochran-Armitage trend test) (Figure 6, Table 4).

**Table 3:** Patient zinc concentrations (Zc) and spouse ages. Spouse ages are shown as median (Q1, Q3). A Steel's test was used. There was no association between spouse age and patients' zinc concentrations.

Patient Zc (μg/dL)	Spouse ages (years)	p-Value
<60	37 (34, 40)	-
60-79	37 (33, 40)	0.96
80-130	36 (32, 40)	0.515
>130	36 (32, 38)	0.44

**Table 4:** Pregnancy rates and patient zinc concentrations (Zc) values and blood drawing time. Confidence intervals for proportions were calculated using the exact binomial Clopper-Pearson method.

AM	Pregnancy rate (%)	95% CI	PM	Pregnancy rate (%)	95% CI
Zc (μg/dL)			Zc (μg/dL)		
<60	65.8	48.6-80.4	<60	52.2	36.9-67.1
60-79	54.0	50.6-57.3	60-79	52.4	48.5-56.2
80-130	58.8	56.5-61.1	80-130	56.6	53.4-59.8
>130	64.0	42.5-82.0	>130	31.6	12.6-56.6

## Discussion

Optimal levels of various essential elements, such as zinc and copper, may affect fertility and male reproduction. Since trace minerals have been currently decreasing in the soil in some areas, the importance of trace elements has been increasing [4]. Zinc is an important trace element that plays an important role in gonadal development, spermatogenesis, and hormone synthesis, and must be ingested as it cannot be produced in the human body. Zinc content accumulates in germ cells over time and increases in the testes during spermatogenesis [5]. Zečević et al. reported that zinc concentrations in seminal plasma need to be maintained at an optimal level, and that low zinc levels are associated with impaired spermatogenesis and general male infertility [6]. Conversely, high zinc levels lead to oxidative stress and other alterations that result in male infertility. Thus, excessively high or low zinc levels in the body impair male reproductive function.

Seminal plasma is known to have the highest Zc value among human tissues, and there is much literature regarding the relationship between Zc and sperm function [7]. On the other hand, serum Zc are also used to study the relationship between Zc and sperm function [1,2,6,8]. Although assessing Zc in semen would certainly be preferable, measuring Zc in semen is not covered by the Japanese health insurance system. It would place a heavy burden on patients. We evaluated Zc in serum rather than in semen.

Zinc and testosterone

Zinc deficiency can cause structural damage to the testes and impaired testosterone synthesis through oxidative stress and autophagy, potentially adversely affecting normal male reproductive development, spermatogenesis, and fertility [1,9]. Since Zc are positively correlated with total testosterone, zinc deficiency may cause reduced testosterone levels. Jalali et al. reported that oral zinc therapy resulted in a significant increase in total testosterone levels in the hemodialysis patients [10]. On the other hand, Koehler et al. concluded that zinc-containing supplements did not alter serum testosterone levels in young healthy males [11]. The effect of zinc on serum testosterone may vary depending on basal zinc and testosterone levels, zinc dosage form, elemental zinc dose, and duration. In a systematic review, Te et al. concluded that zinc-containing medication or supplementation may improve testosterone levels [12]. This study has confirmed that higher zinc levels were associated with increased testosterone levels.

Zinc and semen analysis

Zinc also plays an important role in spermatogenesis and sperm motility; however, controversy exists regarding Zc and semen findings. Excessively high or low Zc levels are associated with impaired male reproductive function. Although numerous studies have demonstrated a correlation between seminal plasma Zc and sperm physiology, it cannot be conclusively determined that zinc deficiency in semen directly causes infertility because there are many causes of infertility [3]. Osadchuk et al. reported that seminal zinc concentrations were closely associated with semen parameters in young men in a population-based study; zinc deficiency may be an important risk factor for lowered semen quality [13]. They emphasized that seminal Zc determinations should be considered a useful tool, in addition to other parameters, for assessing male fertility. Vashisht et al. also reported that seminal plasma Zc were significantly lower in infertile males with asthenozoospermia, non-obstructive azoospermia, and oligozoospermia compared with fertile males in a study of 50 infertile males and 50 age-matched fertile males [14]. We previously found no correlation between serum Zc and sperm count or motility; however, using large-scale data, we found that serum Zc was significantly lower in patients with oligozoospermia and azoospermia than in those with normal semen findings [8]. However, we could not evaluate seminal Zc; serum Zc did not show a positive correlation with semen quality across diagnoses in this study.

Semen findings are well known to vary widely on a daily or weekly basis [15]. Furthermore, a higher sexual abstinence period influenced sperm quality [16,17]. Thus, since semen findings vary widely, it is difficult to determine a baseline value. In this study, semen analysis was evaluated only once, and the period of abstinence was not considered. These points can be considered as limitations of this study.

Zinc and medication or supplement taken

Zinc deficiency can cause infertility even in patients without physical abnormalities or symptoms. However, there are no oral medications or supplements that are scientifically proven to improve semen quality. Wong et al. enrolled 103 infertile males and 108 fertile males and administered the same dose of zinc for the same duration. They conducted a double-blind, randomized, placebo-controlled study to evaluate the effects of zinc 5 mg/day with folic acid [18]. They reported that in both infertile and fertile males, sperm counts increased from approximately 7.5×10^6^/mL before treatment to 12×10^6^/mL after treatment, with no significant differences observed in other semen parameters [19]. In the current study, no significant differences in Zc were observed among patients who took or did not take medication, smoked, or took supplements.

Smoking and androgenic alopecia (AGA) medication

It has long been reported that smoking habits can worsen sperm quality and reduce pregnancy rates. Osadchuk et al. reported that heavy smokers were characterized by a decreased semen volume, total sperm count, sperm concentration, and motility. They also showed reduced Zc in serum and seminal fluid. However, no significant differences between smokers and non-smokers were found for serum LH, FSH, and testosterone levels [20]. Holmboe et al. reported that not only cigarette smoking but also the use of e-cigarettes is associated with lower sperm count [21]. They emphasized that even e-cigarettes may be harmful to males trying to achieve a pregnancy, despite the fact that they are often considered to be less harmful than conventional cigarette smoking.

All sperm parameters, including sperm quantity, density, motility, and morphology, were significantly lower in participants with moderate to severe androgenetic alopecia (AGA) than in participants with normal to mild AGA [22]. Santana et al. reported that AGA treatment drugs can adversely affect the testes and testis-derived cell cultures in mouse models [23]. Finasteride can damage the epididymis and reduce sperm quality. However, due to the lack of research on the effects of minoxidil on the epididymis, it is impossible to determine whether this drug also affects this organ. They concluded that both drugs act through mechanisms involving hormonal disruption and oxidative stress, causing morphological changes that disrupt normal testicular function, potentially leading to reduced sperm quality and reduced fertility [23].

In this study, the number of patients receiving oral AGA medication was small, and the effects on semen findings and ART pregnancy were unknown. Our recommendation is that AGA medications may also contribute to worsening semen findings, but, similar to smoking, it is wise to avoid them if poor semen findings are present.

Blood collection time

Because zinc levels are known to decrease from evening to night, blood should generally be collected early in the morning on an empty stomach. Killilea and Schultz reported that even when the same blood samples were collected from the same donor, significant differences in zinc content were observed depending on the blood draw site, blood sample matrix, blood processing time, and blood holding temperature [24]. Even with the same donors and blood samples, zinc results varied significantly depending on the draw site, collection tube, and processing method, indicating that pre-analytical factors can materially affect zinc measurements and the classification of zinc status. In our study, all samples were venous serum, processed within 30 minutes, and kept at 4°C, so these conditions were unlikely to significantly affect Zc.

Berti et al. reported that children whose blood samples were collected in the morning without fasting had lower Zc than those whose samples were collected in the afternoon, whereas those whose samples were collected in the morning while fasting had higher Zc [25]. The difference in adjusted Zc by collection time and fasting state was significant in the upper percentiles of the distribution, with the largest absolute difference observed between children whose blood samples were collected in the morning and those whose blood samples were collected in the afternoon. In this study, differences in Zc were observed across collection times, suggesting caution when evaluating Zc values. Zc in the general population are lower later in the day. This study also confirmed that blood drawing in the afternoon resulted in lower Zc.

Zinc and ART outcomes

Spouse age is very important when evaluating pregnancy outcomes. Low or high Zc may have a negative impact on ART pregnancy outcome. However, the extent to which Zc affect pregnancy outcomes has not been specifically reported.

There was no significant difference between the patient Zc and the spouse's age, and Zc did not affect pregnancy outcome in this study. Although overall pregnancy rates tended to decline with Zc increasing, when pregnancy rates were examined by dividing Zc drawing time into AM and PM, no significant differences were observed. No significant differences in Zc or pregnancy rates among partners were observed with the intake of various oral medications or supplements. Supplements seem not to result in high serum zinc levels or affect pregnancy outcomes.

Limitations and reasons for caution

Zc was assessed using serum values rather than seminal plasma. Semen findings fluctuate widely on a daily or weekly basis and also depend on the abstinence period. This was a cross-sectional, retrospective study using a single semen analysis without considering the abstinence date.

Wider implications of the findings

Since zinc is an important trace element, many male infertility patients take empiric oral medications and/or zinc-containing supplements. No associations were observed between Zc and semen findings or pregnancy outcomes using ART.

## Conclusions

Zinc plays an important role in hormone production and semen quality, and we reported that Zc were significantly lower in azoospermic patients compared with normal semen findings. However, this large cross-sectional study did not find a positive correlation between serum Zc and sperm parameters or pregnancy rate, despite increased testosterone levels. Smoking, oral medication, or using supplements did not impact Zc. Further accumulating cases and evaluations are needed.
